# Natural Dyes and Antioxidant Compounds from Safflower (*Carthamus tinctorius* L.) Florets: The Effects of Genotype and Sowing Time

**DOI:** 10.3390/plants15020282

**Published:** 2026-01-17

**Authors:** Clarissa Clemente, Silvia Tavarini, Shaula Antoni, Silvia Zublena, Luciana G. Angelini, Ilaria Degano

**Affiliations:** 1 Department of Agriculture, Food and Environment, University of Pisa, Via del Borghetto 80, 56124 Pisa, Italy; clarissa.clemente@agr.unipi.it (C.C.); luciana.angelini@unipi.it (L.G.A.); 2 Interdipartimental Research Center “Nutraceuticals and Food for Health”, University of Pisa, Via del Borghetto 80, 56124 Pisa, Italy; 3 Department of Chemistry and Industrial Chemistry, University of Pisa, Via Moruzzi, 13, 56124 Pisa, Italy; shaulaantoni@gmail.com (S.A.); ilaria.degano@unipi.it (I.D.); 4 Pisa Botanic Garden and Museum, University of Pisa, Via Luca Ghini 13, I, 56126 Pisa, Italy; silvia.zublena@unipi.it

**Keywords:** bioactive pigments, phenolic compounds, genotypic variability

## Abstract

Safflower (*Carthamus tinctorius* L.) is increasingly attracting the attention of Mediterranean farmers due to its broad environmental adaptability and low input requirements. Although still relatively underexplored, this species holds remarkable potential as a source of natural dyes and bioactive phytochemicals with recognized health-promoting and phytotherapeutic properties. In this study, the effects of genotype and sowing time on safflower’s productive and qualitative traits were investigated by testing six genotypes and two sowing times (autumn and spring) in an open-field trial conducted in central Tuscany. The Pieve genotype achieved the highest floret dry yield per head, number of heads per plant, and total floret yield per plant, whereas the Montola 2000 genotype was distinguished by its elevated polyphenol concentration and pronounced antioxidant activity. Autumn sowing resulted in higher yields of bioactive pigments, including carthamin and yellow quinochalcones, alongside greater total phenolic content and antioxidant capacity. Conversely, spring sowing appeared to limit pigment biosynthesis, likely due to environmental stressors such as elevated temperature and excessive light exposure. Overall, these findings highlight the strong influence of genotype and sowing time on the accumulation of health-beneficial compounds in safflower. By optimizing these factors, safflower can be strategically valorized as a multipurpose crop in the Mediterranean region, combining economic and environmental sustainability with the production of natural compounds of high nutraceutical and phytotherapeutic value.

## 1. Introduction

Safflower (*Carthamus tinctorius* L., Family Asteraceae), a versatile oilseed crop, may offer multiple benefits for cereal-based cropping systems due to its notable competitiveness against weeds and resistance to cold, drought, salinity, and bird predation, as well as its reduced consumption of chemicals and water; thus, it is more likely than major crops to succeed under global climate change [1,2]. Nowadays, safflower is gaining popularity worldwide as a multipurpose crop due to the large amount of products and co-products that can be derived from it. In fact, the full use of safflower biomass through the recovery/valorization of all of its components through a cascade biorefinery approach could provide a range of eco-friendly bio-based products with high added value [3,4]. Safflower is known for its high-quality edible oil rich in polyunsaturated acids and for the pigments extracted from its florets, which have been used for years in Traditional Chinese Medicine as natural dyes in foods, cosmetic formulations, and textiles [5,6]. Brunello [7] reports that the crop probably originated in Western or Southern Asia and was indeed documented in ancient Egyptian texts from the VI dynasty, with traces found in Theban tombs (2000 BCE). Its use spread to ancient Greece, where it was noted by Aristotle, Theophrastus, and Galen, and is even referenced in the Talmud [8]. Cultivation extended to India and China, where it was introduced via the Silk Road around the 3rd century AD and called hong hua (紅花), meaning “red flowers” [9]. Traces of safflower dyes have been found in Chinese textiles from the 10th to 17th centuries [10] and in Dunhuang fabrics (7th–10th centuries) [11]. In Europe, safflower dyeing was practiced since at least the 16th century, with carthamin detected in an orange sample from a Florentine tapestry [12] and in historical textiles originally from Italy or Russia, now belonging to the Victoria and Albert Museum and currently stored at Blythe House, London [13]. Dried safflower petals contain yellow and red quinochalcone natural dyes [14]; in fact, safflower yellows are amongst the few water-soluble yellow dyes found in nature [15], while the red pigment is water-insoluble. The major components of safflower yellows are the chalcones hydroxysafflor yellow A and safflor yellow B, which are present alongside some other minor components, such as pre-carthamin, safflor yellow A, anhydrosafflor yellow B, safflomin A, safflomin C, isosafflomin C, tinctormine, cartormin, apigenin, and kaempferol [16,17,18,19,20]. Carthamin, which exhibits antioxidant activity, is the major molecule responsible for red coloring in safflower [21,22]. These chalcones are the main constituents of glycosylated flavonoids in safflower; notably, they have not been detected in other natural products. They exhibit broad biological activities [20,23,24,25], including anti-oxidant, -inflammatory, -cancer, and -hypertension effects; blood circulation-promoting effects; and hepatic and neuroprotective properties [20,23,26,27,28]. Natural dyes are particularly relevant today because of their minimal environmental and health impacts compared to synthetic dyes, which have been suggested as contributors to health issues such as skin cancer and allergic contact dermatitis [29,30]. Nevertheless, the production of these natural colorants is strongly affected by many extrinsic and intrinsic factors such as the genotype, biotic and abiotic factors, ontogenetic stage, and growing region [1,31]. Among these factors, the sowing time plays an essential role in secondary metabolite production [32]; however, very few studies are available on the impact of both the sowing time and genetic characteristics on floret production and secondary metabolite accumulation [32,33]. Thus, the aim of this study was to define the optimal conditions for maximizing the levels of beneficial bioactive compounds and antioxidant content of safflower petals derived from six genotypes. Therefore, the effects of genotype and sowing time on the yield and accumulation of yellow and red dyes, total phenols, flavonoids, and antioxidant capacity were evaluated in safflower genotypes grown in Mediterranean environments.

## 2. Results

### 2.1. Weather Conditions

**Figure 1:**
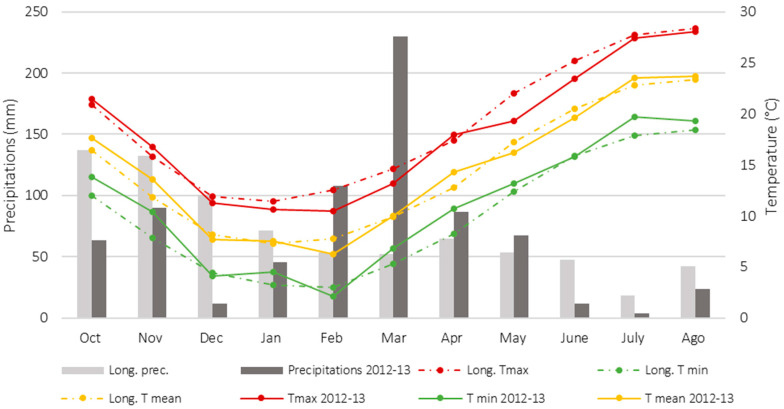
Meteorological data from the long-term period (1992–2011) and 2012–2013 growing season at the Experimental Centre of DAFE, University of Pisa (San Piero a Grado, Pisa, Italy 43°40′ N; 10°19′ E).

Figure 1 shows the monthly meteorological conditions of the 2012–2013 growing season (from October to August), obtained from an automatic station located near the experimental site. The cultivation site is characterized by a Mediterranean climate, with the minimum low temperature occurring in February (mean monthly value of 2 °C), the maximum high temperature occurring in August (mean monthly value of 28 °C), and about 765.4 mm of cumulative rainfall from October to August. During summer (July–halfway through August), a dry period generally occurs with low rainfall and high air temperatures. The growing season is characterized by more intense rainfalls than those recorded in the long-term period, especially in the period from February to May, when a peak of 250 mm of rainfall was recorded in March. In contrast, the air temperatures during the 2012–2013 growing season were essentially in line with those during the long-term period.

### 2.2. Surveyed Parameters at Harvest

Our results revealed a significant effect of genotype (G) and sowing time (S) on floret yield per head, number of heads per plant, and floret yield per plant, while no effect from a G × S interaction was detected (Table 1). The Pieve genotype was characterized by the highest floret yield per head, the highest floret yield per plant, and also the highest head number per plant; conversely, the Roberto genotype showed both the lowest number of heads per plant (−49.6%) and floret dry yield per plant (−56.8%). Regarding the sowing time, an increase in the floret dry yield per head (+16.7%) was observed when sowing was performed in spring, while the number of heads per plant significantly decreased after moving the sowing time from autumn to spring (−14.3%); conversely, no significant differences were observed on the floret dry yield per plant both in autumn and spring sowing (Table 1).

### 2.3. Safflower Quality: Red and Yellow Components

**Figure 2:**
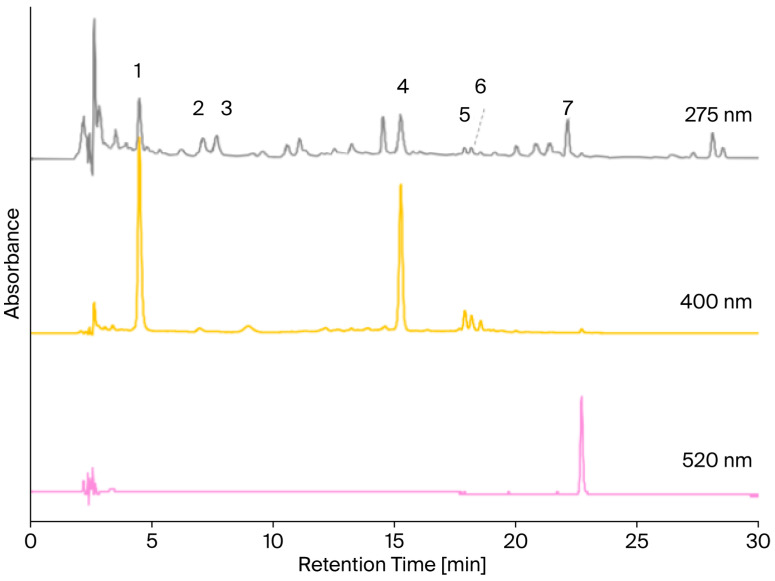
Chromatograms at 275, 400, and 520 nm of the extract derived from the Boemondo sample sown in autumn (not to scale). Peak 1: hydroxysafflor yellow A; peaks 2 and 3: flavonoid glucosides; peak 4: safflor yellow A; peak 5: safflomin C; peak 6: isomer of safflomin C; peak 7: carthamin.

All samples were extracted and analyzed to determine the profile of the main dyes present. The identified compounds, along with their spectroscopic and spectrometric characteristics, are reported in Table 2, while Figure 2 shows an example of the chromatograms at different wavelengths (275, 400, and 520 nm, respectively) obtained from the extract from the Boemondo genotype sown in autumn. Specifically, hydroxysafflor yellow A, flavonoid glucosides, safflor yellow A, safflomin C, isomer of safflomin C, and carthamin were identified. Representative chromatograms for each genotype and sowing time are reported in the Supplementary Materials (2. Supplementary Figures, Figures S1–S6). The amount of carthamin in the different samples was evaluated using calibration curves employing an analytical standard of this compound. Regarding the quinochalcones, for which commercial standards were not available, peak areas were used as proxies for analyte concentration, and a semi-quantitative comparison within the sample set was performed (see Section 4.4). These data, although very useful for comparing samples with one another, cannot be used to evaluate the different amounts of each yellow quinochalcone within one sample, since different compounds have different molar absorptivity and thus their relative response at the chosen integration wavelength might be different. Notwithstanding this, the semi-quantitative analysis allowed us to compare the results obtained from the different samples; a significant effect of sowing time and genotype on the quinochalcone and carthamin content was observed. The results were obtained by performing extractions from the petals in their original or powdered form; first of all, the results obtained for the same genotype and sowing time were compared, showing that the extraction yields were not statistically different at a 95% confidence level.

**Figure 3:**
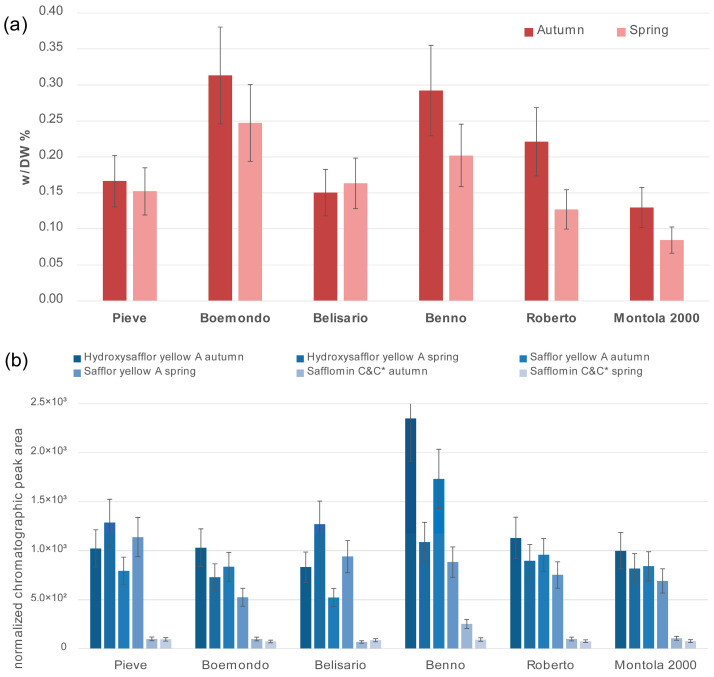
(**a**) The carthamin content (% of petal dry weight) extracted from each genotype at the two sowing times (the amount is reported in w/DW %). (**b**) The peak areas (integrated in the chromatogram at 400 nm and normalized towards sample weight and extraction solvent weight) of each quinochalcone, extracted from each genotype on the two sowing dates. C* refers to the safflomin C isomer. Error bars represent 95% confidence interval for triplicate measurements.

The carthamin yields (% of petal dry weight) for each genotype sown in autumn and spring are reported in Figure 3. The *T*-test performed on the two datasets highlighted that for the Pieve and Belisario genotypes, no significant differences in the carthamin content (c.a. 0.15%) were obtained for either autumn or spring sowing; meanwhile, for the other genotypes, the safflower plants sown in autumn showed a significantly higher carthamin content compared to those sown in spring. The Benno and Boemondo genotypes sown in autumn showed the highest carthamin content, which was around 0.3%, while Montola 2000 seemed to produce the lowest amount of this pigment when sown in both autumn and spring.

A significant effect of sowing time and genotype (at *p* ≤ 0.05, according to Fisher’s LSD test) was observed on the content of yellow quinochalcone compounds for Belisario and Benno, as reported in Table 3/Figure 3b. Notably, the evaluation was performed on peak areas normalized towards sample weight; thus, it does not provide absolute values and can only be used for comparison amongst the set of samples. The sowing time affected the production of the yellow quinochalcone compound hydroxysafflor yellow A and safflor yellow A since the normalized peak areas, obtained for autumn and spring, were significantly different in both genotypes. The relative amount of the sum of safflomin C and its isomer is also different in Benno genotype sown in autumn and spring, while that of Safflor yellow A shows a difference in Pieve genotype sown in autumn and spring. In general, the genotype Benno sown in autumn showed the highest amount of yellow dyes, while the Belisario genotype exhibited the highest amount when sown in spring. Finally, the Benno genotype sown in autumn featured the highest relative amount of yellow quinochalcones among the genotypes.

### 2.4. Polyphenols and Antioxidant Activity

Genotype (G), sowing time (S), and their interaction (G × S) significantly affected the total phenol content, antioxidant capacity (measured by FRAP assay), and anti-radical activity (measured by DPPH assay), but did not affect the total flavonoid content, which did not change depending on sowing time (Table 4). Furthermore, the block effect was not significant for the antioxidant traits considered.

In particular, the total phenol content was higher in the autumn-sown plants (+14.7%) compared to the spring-sown plants. According to the G × S interactions, among genotypes, Montola 2000 showed the highest total phenol content in autumn sowing, followed by Pieve, Belisario, and Roberto; the opposite trend was observed in spring, with the lowest value obtained in the Pieve, Belisario, and Boemondo genotypes (Table 5). The total flavonoid content was also significantly affected by G and the G × S interaction, with the highest content recorded for the Montola 2000 genotype sown in spring, while the lowest contents were obtained for the other genotypes when sown in both autumn and spring, with an average total flavonoid content of about 3.94 mg GAE g^−1^ (Table 6).

Furthermore, in order to evaluate the total antioxidant capacity, both the FRAP and DPPH assays were performed. A significant effect of G and S and their G × S interaction was observed, as reported in Table 7 and Table 8, respectively. The total antioxidant capacity, measured using the FRAP method, was significantly affected by the sowing time, with higher mean values obtained in the safflower florets collected from plants sown in autumn (+14.6%). Among the genotypes, Montola 2000 showed the highest antioxidant activity, particularly when sown in autumn, followed by Belisario and Pieve. The latter, however, showed the lowest values when sown in spring (Table 7). The DPPH radical-scavenging activity was determined as a component of the total antioxidant activity expression; the IC_50_ values were similar between the plants sown in autumn and spring, although a slight but significant increase was observed in the spring-sown plants (+4.1%) compared to the autumn-sown plants (Table 8). According to the G × S interaction, spring-sown Pieve-genotype plants, characterized by the highest IC_50_ values, exhibited weaker antiradical activity, whereas autumn-sown Montola 2000, characterized by the lowest IC_50_ values, showed the strongest antiradical activity (Table 8).

### 2.5. Principal Component and Hierarchical Cluster Analyses on Safflower Quality

**Figure 4:**
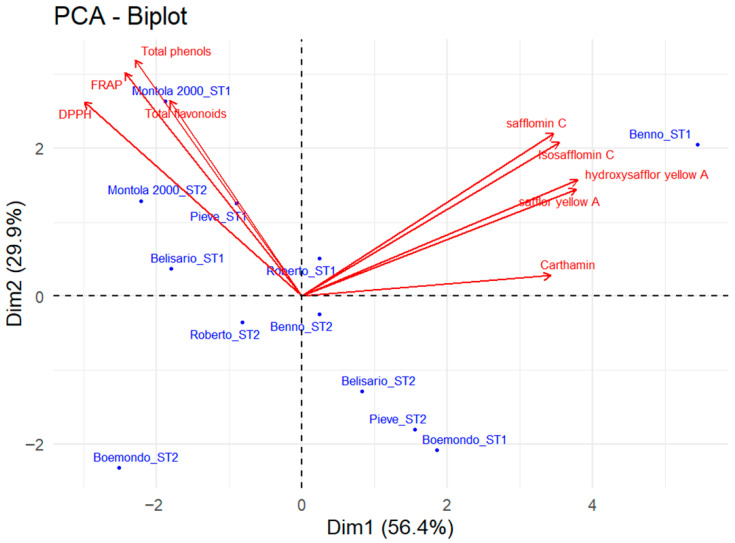
PCA biplot (score plot + loading plot) based on Dim1 (PC1) and Dim2 (PC2) describing the variability in yellow and red pigments (safflor yellow A, hydroxysafflor yellow A, safflomin C, isosafflomin C, and carthamin) and antioxidant traits (total phenols, total flavonoids, FRAP, and DPPH) among safflower genotypes in the two sowing times (ST1 = autumn; ST2 = spring).

In order to further investigate the genotype effects impacting safflower quality, a PCA was carried out to highlight factors possibly correlating with qualitative safflower characteristics, and to then identify clusters across genotypes and sowing times. The PCA biplot (Figure 4) explained 86.3% of the total variance, with PC1 and PC2 accounting for 56.4% and 29.9%, respectively. The loadings of the variables associated with each principal component are reported in the Supplementary Materials (3. Supplementary Tables, Table S3). PC1 primarily discriminated samples according to pigment-related compounds, including hydroxysafflor yellow A, safflor yellow A, safflomin C, isosafflomin C, and carthamin, which showed high positive loadings along this axis. In contrast, PC2 was mainly represented by antioxidant-related traits (total phenols, total flavonoids, FRAP, and DPPH) and was negatively associated with PC1. Based on *the* PCA biplot, the identification of three main groupings based on the similarity in their content of yellow and red components and antioxidant-related traits was obtained. The first group was represented by the samples located on the positive side of PC1, characterized by higher contents of yellow and red pigments, and primarily included the Benno_ST1 genotype sown in autumn sowing. A second group, positioned on the negative side of PC1 and positive along PC2, comprised genotypes with higher antioxidant capacity, notably Montola 2000, both in autumn (ST1) and spring sowing (ST2), together with Pieve_ST1, Belisario_ST1, Benno_ST1, and Roberto_ST1 and ST2. Finally, a third group, distributed mainly in the lower quadrants of the biplot, included samples showing intermediate to lower values across most qualitative variables, such as Pieve_ST2 and Belisario_ST2, and Boemondo, both in autumn and spring sowing.

**Figure 5:**
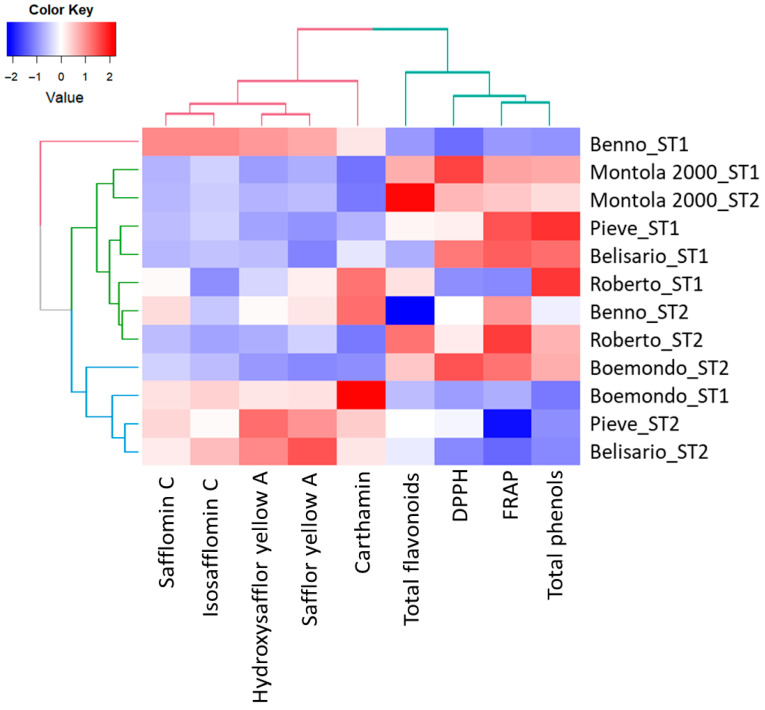
Hierarchically clustered heatmap of safflower quality according to genotype and sowing time (ST1 = autumn; ST2 = spring). Data values were transformed to color scale.

These observations were confirmed by the two-way dendrogram (HCA) reported in Figure 5. Through the heatmap representation, it is possible to simultaneously visualize the clusters of samples (genotypes) and variables (=red and yellow components, total phenols, total flavonoids, and antioxidant activity measured by FRAP and DPPH) and to find the variables that appeared to be characteristic for each sample cluster. Based on the HCA, three main macro-clusters of samples were identified, each further subdivided into subgroups. The first macro-cluster (red) comprised only Benno_ST1, which was characterized by the highest levels of red and yellow components. Montola 2000*_*ST1 and ST2*,* Pieve_ST1, Belisario_ST1, Roberto_ST1 and ST2, and Benno_ST2, characterized by the lowest values of red and yellow components and, conversely, by a high–intermediate relative total flavonoids, total phenols, and antioxidant activity measured through FRAP and DPPH. Finally, the third macro-cluster (blue) represented by the genotypes Boemondo ST1 and ST2, Pieve_ST2, and Belisario_ST2 showed intermediate to low values across most variables, except for a few showing moderate enrichment in components such as DPPH, hydroxysafflor yellow A, safflor yellow A, and carthamin. Overall, the hierarchically clustered heatmap showed a clear separation among the genotypes determined by the relative abundance of each qualitative variable considered. The formation of subgroups was mainly due to high, intermediate, or low levels of red and yellow components, total phenols, total flavonoids, and the related antioxidant activity measured using FRAP and DPPH.

## 3. Discussion

In recent years, there has been growing interest in alternative crops, like safflower, particularly within the framework of sustainable agriculture and bioeconomy. Safflower is a versatile and sustainable crop that offers significant opportunities for crop diversification, reducing environmental impact, and driving innovation across sectors such as food, cosmetics, and nutraceuticals. Although our experiment was conducted before the most recent literature was published, the few current studies on safflower in Mediterranean environments have mainly focused on individual cultivars, often emphasizing productivity or individual bioactive constituents. In contrast, our study (i) highlighted the strong genotype-dependent variability in the bioactive compounds observed according to the sowing time, and (ii) provided a qualitative characterization of red and yellow quinochalcones and bioactive compounds with high antioxidant capacity, such as phenols and flavonoids. In this regard, we aimed to provide information on the introduction of safflower in the typical Mediterranean climate as a suitable and innovative crop by comparing six varieties and two sowing times. As a general trend, our results showed that the head and floret yields of safflower were significantly affected both by genotype and sowing time; in particular, the genotype Pieve was characterized by the highest floret yield (both per head and per plant) and head number per plant; when sown in spring, an increase in the floret dry yield per head was observed, while the number of heads per plant significantly decreased when moving from autumn to spring sowing. Consistently, in a study carried out in Southern Italy, Patanè et al. [33] observed that the sowing time had a greater impact on head and floret productivity in safflower. In particular, these authors found that, when the sowing time was shifted from late February to late April, a progressive decrease in the total number of flower heads occurred as a consequence of the reduced number and size of flower heads, probably due to water and light limitations. On the contrary, in our environmental conditions, regardless of variety, a substantial increase in floret yield per head was noted; however, this was accompanied by a reduction in the head number per plant. At the same time, it is important to underline that the floret yield also strictly depends on the harvest time. In this regard, when comparing different floret harvest times (onset of flowering, after pollination, onset of petal wilting), Mohammadi and Tavakoli [34] found that for spring safflower varieties, the highest floret yields were achieved at the onset of flowering. Moreover, from a qualitative point of view, a significant effect of sowing time and genetic characteristics on yellow quinochalcones and carthamin content was recorded, with a similar concentration of the latter (ranging 0.02–0.88% by weight) to that reported by other authors [33,35,36]. Our findings highlighted that autumn sowings resulted in a higher content of safflomins (safflomin C and isosafflomin C) and carthamin in the florets. In particular, for the Pieve and Belisario genotypes, no significant differences in the carthamin content (c.a. 0.15%) of the extracts derived from both the autumn- and spring-sown plants were observed, while for the other four genotypes, the safflower plants sown in autumn showed a significantly higher carthamin content compared to those sown in spring. The Benno and Boemondo genotypes, sown in autumn, showed the highest carthamin content, reaching 0.30%. Thus, spring sowing appeared to negatively influence the biosynthesis or accelerate the degradation of pigments in safflower florets; this observation aligns with previous research indicating that natural colorants are highly susceptible to environmental factors such as temperature, light, and pH [37,38]. Notably, carthamin has been shown to be particularly unstable, more than yellow pigments, especially under conditions of high temperature and prolonged daylight exposure [39]. A significant effect of sowing time and genotype was also observed on the content of yellow quinochalcone compounds. In particular, each genotype showed a different profile when sown in autumn and spring, with the exception of Boemondo and Montola, which did not present evident differences in their qualitative profiles. In this framework, the higher levels of quinochalcones and phenolic compounds observed with autumn sowing can be interpreted in light of flavonoid biosynthesis being strictly regulated by various environmental factors such as temperature, light, and oxidative stress [40,41,42,43]. Flavonoid biosynthesis is regulated by a coordinated network of structural enzymes (e.g., PAL—phenylalanine ammonia-lyase, CHS—chalcone synthase, CHI—chalcone isomerase, F3H—flavanone 3-hydroxylase, and downstream hydroxylases such as F3′H—flavonoid 3′-hydroxylase and F3′5′H—flavonoid 3′,5′-hydroxylase) and glycosyltransferases, the transcription of which is strongly influenced by temperature and light through MYB–bHLH–WDR transcriptional complexes [43,44,45]. Under the environmental conditions of our study location, the cooler temperatures and moderate irradiance typical of the autumn–winter period likely promoted the activation of flavonoid biosynthetic pathways, as well as the stabilization of quinochalcone structures. In contrast, spring sowing exposed the safflower plants to higher temperatures and intense light during the flowering stage, conditions that not only may have enhanced pigment photo-oxidation and thermal degradation—thereby reducing the net accumulation of carthamin and yellow quinochalcones—but also limited the effective build-up of these pigments, which, despite the induction of several flavonoid-related genes by developmental and stress signals, remain highly susceptible to environmental factors.

To date, more than 200 compounds have been identified and isolated from safflower, including quinochalcones, phenols, flavonoids, alkaloids, and aromatic glucosides [46,47,48]. Their concentration mainly depends on factors such as genotype, environmental conditions, and also on the extraction technique [49]. Djeridane et al. [50] showed a significant correlation between warm temperature, high sun exposure, drought, salinity, and activation of plant defense mechanisms resulting in secondary metabolite biosynthesis. Additionally, Salem et al. [51] showed how the use of pure solvents (i.e., ethanol) for polyphenols extraction led to the lowest phenolic content. On the contrary, aqueous solvent extracts provide the highest content, considering that hydrophilic compounds require a small amount of water to be extracted [52]. Our results, for both total phenols and total flavonoids, are in agreement with those described by Salem et al. [31,51], who reported values between 7 and 31 mg GAE g^−1^ dry weight of total phenols and between 2 and 7 mg CAE g^−1^ dry weight of total flavonoids, depending on the stage of flower development and color. More specifically, these authors observed how variability in the total phenolic content strongly correlated with the flower and, hence, petal color, with the lowest phenolic content in yellow flowers, followed by red and orange flowers, which featured the highest phenolic content values. It should also be noted that the slight differences found between these data could be justified by the fact that the genotypes selected for our study were oilseed varieties, which are not specifically selected for dyeing purposes. In addition, these secondary metabolites represent natural antioxidants capable of exhibiting a wide range of biological activities, including radical-scavenging, anti-inflammatory, vasodilatory, antithrombotic, and neuroprotective and hepatoprotective effects [26,53,54,55]. For instance, hydroxysafflor yellow A has been reported to promote vasodilatation of pulmonary arteries and to improve microcirculation and protection against ischemia–reperfusion injury, while saffloflavone and other flavonol glycosides isolated from safflower flowers display cardioprotective effects [56]. Carthamin is also known for its antiulcer, antihistamine, anti-inflammatory, antimicrobial, and cytotoxic abilities [46], as well as its coloring properties, which are exploited in the food, textile, and cosmetic industries. Furthermore, our results highlighted a clear genotype-dependent variability in the scavenging activity of the safflower flowers, underscoring the importance of genotype selection. The IC_50_ values obtained in this study were higher than those reported by previous studies; for instance, Salem et al. [31,51], comparing safflower flowers of different colors, reported IC_50_ values ranging from 2.50 to 5.00 μg mL^−1^ depending on the flower developmental stage. However, it should be noted that comparisons across studies are challenging, as differences in extraction methods and modifications of DPPH assay protocols can strongly influence the measured antiradical activity [32]. Among the genotypes evaluated, Montola 2000, the only high-oleic variety tested, showed the most favorable qualitative profile, while Boemondo exhibited the lowest antioxidant activity and the lowest levels of bioactive compounds. Furthermore, as suggested by the PCA biplot, differences in bioactive compounds and pigment composition of safflower were mainly determined by the genotype, while sowing time acted as a secondary factor modulating these responses within each genotype. The PCA biplot highlighted a clear separation between pigment-related compounds (red and yellow components) and antioxidant-related traits (total phenols, total flavonoids, FRAP, and DPPH), indicating different patterns of metabolite accumulation among genotypes. In particular, genotypes such as Benno_ST1 were mainly associated with higher contents of yellow and red pigments, whereas Montola 2000_ST1 showed a stronger association with antioxidant-related traits, including total phenols, total flavonoids, FRAP, and DPPH. Other genotypes, including Pieve, Belisario, Roberto, and Boemondo, showed variable responses depending on sowing time, indicating that environmental conditions could modulate genotype-specific qualitative traits. These results were further supported by HCA, which identified three sample groupings in line with the PCA results. Finally, genotype selection plays a key role in dual-purpose safflower cultivation, as it influences both the floret yield and the accumulation of high-value secondary metabolites. Genotypes that combine good agronomic performance with high concentrations of target bioactive compounds provide greater opportunities for valorization within high-value supply chains, including nutraceuticals, natural colorants, and cosmetic ingredients. In addition, genotypes with richer metabolite profiles require less raw material to achieve a given extract yield, which lowers processing costs and ensures more consistent product quality. Overall, these aspects highlight the importance of selecting genotypes based on both yield and qualitative traits to enhance the economic viability of safflower production.

## 4. Materials and Methods

### 4.1. Chemicals

For sample treatment, the solvents used were dimethylformamide (DMF) (Sigma Aldrich, Saint Louis, MO, USA), bi-distilled water (H_2_O, Carlo Erba, Milan, Italy), methanol (MeOH, HPLC-grade, Sigma Aldrich, USA), and ethanol (EtOH, HPLC-grade, Sigma Aldrich). The eluents used for the HPLC-DAD system were bi-distilled water (Carlo Erba, Milan, Italy), acetonitrile (ACN, HPLC-grade, Sigma Aldrich, USA), and trifluoroacetic acid (Sigma Aldrich, USA). The eluents used for the HPLC-ESI-Q-ToF system were water, acetonitrile (LC-MS grade, Sigma- Aldrich, USA), and formic acid (Sigma-Aldrich, USA). Carthamin was purchased from Apin Chemicals LTD (Oxon, UK). A 500 μg g^−1^ stock 94 solution was prepared in DMF and stored in the dark at −18 °C. All stock solutions of flavonoids were prepared in methanol and stored in the dark at −18 °C. Working solutions for calibration curves were prepared in water for carthamin and in methanol for flavonoids, both in the range of 0.1–10 μg g^−1^. Once prepared, working solutions were stored in the dark at −4 °C. DPPH (2,2-diphenyl-1-picrylhydrazyl), gallic acid monohydrate (3,4,5-trihydroxybenzoic acid), TPTZ (2,4,6-tri(2-pyridyl)-s-triazine), Trizma acetate, Folin–Ciocalteu reagent, sodium carbonate, and ferric chloride were obtained from Sigma-Aldrich Chemical Co., Ltd. (Milan, Italy).

### 4.2. Site Description and Field Experiment

Six safflower genotypes (Pieve, Boemondo, Belisario, Benno, Roberto, and Montola 2000) were compared in an open-field experiment in which sowing was performed in both autumn and spring during the 2012–2013 growing season at the Experimental Centre for Agro-Environmental Research “E. Avanzi” of the University of Pisa (San Piero a Grado, Pisa, Italy 43°40′ N; 10°19′ E; 5 m elevation), as reported in Abou-Chehade et al. [57]. Safflower seeds were kindly supplied by Prof. E. Alba (University of Basilicata, Italy). The autumn sowing was performed on 25 October 2012, while the spring sowing was performed on 18 April 2013. The morphology of the region was flat, and the soil was an alluvial deep loam, typical of the lower Arno River plain, classified as Typic Xerofluvent according to the USDA system (Soil Survey Staff, 1975). The physical and chemical soil characteristics were evaluated at the beginning of the experiment at a depth of 30 cm. The soil was loam (35.7% sand; 47.6% silt; 16.7% clay), with a medium level of total nitrogen (1.23 mg kg^−1^) and low phosphorus availability (4.53 mg kg^−1^). Additionally, it showed a good average organic matter content (2.02%) with a neutral reaction (pH 7.7). Total nitrogen was evaluated using the macro-Kjeldahl digestion procedure [58], while available phosphorus was assessed by colorimetric analysis following the Olsen method [59], and the exchangeable potassium was determined using the Thomas method [60]. Moreover, soil organic matter content was determined using a slightly modified Walkley–Black wet combustion method [61] and soil pH was measured in a 1:2.5 soil–water suspension [62]. The experimental field had previously been cultivated with durum wheat (*Triticum durum* Desf.). The experiments were arranged in a randomized complete block design with four replications. Plot size was 12 m^2^ (4 × 3 m) and plant density was about 40 plant m^−2^, with an inter-row spacing of 0.5 m. After sowing, plots received 80 kg ha^−1^ of N (in the form of ammonium nitrate), split across two applications. Lastly, plants were grown without supplemental irrigation.

### 4.3. Plant Sampling and Measurements

Safflower capitula were harvested on a 1 m^2^ sampling area in each plot for each genotype when more than 90% of the florets were open (stage 69), according to the method described by Flemmer et al. [63]. The number of capitula (heads) was counted, and then the florets were separated from the head and weighed to determine fresh weight. The florets were air-dried in the dark at a constant 20 °C temperature, in order to evaluate the floret dry yield per plant. Then, they were ground to a fine powder by an electric ball-mill (Fritsch pulverisette 23, Fritsch, Idar-Oberstein, Germany) and stored in a desiccator at room temperature (∼20 °C) in darkness for subsequent analyses.

### 4.4. Determination of Red and Yellow Components

HPLC-DAD analyses were carried out using a HPLC-DAD system equipped with a PU2089 quaternary pump with a degasser and an MD-2010 diode array detector, all Jasco Int. (Tokyo, Japan). The detector operated in the range 200 to 650 nm and had 4 nm resolution. The working wavelengths were 275 nm, 400 nm, and 520 nm. The column was a reverse phase Agilent TC-C18 (particle size 5 μm, 250 × 4.6 mm), with an Agilent TC-C18 precolumn (4.6 × 12.5 mm), both from Agilent Technologies (Santa Clara, CA, USA). Optimal separation was achieved using a gradient elution of 0.1% trifluoroacetic acid in H_2_O (eluent A) and 0.1% trifluoroacetic acid in acetonitrile (eluent B) at a 1 mL min^−1^ flow rate: 15% of B for 5 min followed by a linear gradient to 50% B from 5 to 30 min; then, a linear gradient to 100% B in 5 min, which was held for 5 min. Re-equilibration took 10 min. Injection volume was 30 μL for each sample. HPLC-ESI-Q-ToF analyses were carried out using a 1260 Infinity HPLC, coupled with a 6530 Infinity Quadrupole-Time-of-Flight tandem mass spectrometer via a Jet Stream ESI interface, all Agilent Technologies (Santa Clara, CA, USA) . The HPLC conditions were as follows: Zorbax-C18-extended column (particle size 1.8 μm, 50 mm × 2.1 mm) with a Zorbax Eclipse Plus C18 Analytical Guard Column (particle size 5 μm, 4.6 mm × 12.5 mm), both Agilent Technologies (Santa Clara, CA, USA). The flow rate was 0.2 mL min^−1^, injection volume was 4 μL for each sample. The optimal separation was achieved using a gradient elution of 1% formic acid in H_2_O (eluent A) and 1% formic acid in acetonitrile (eluent B): linear gradient from 15% B to 50% B in 6 min, followed by a linear gradient to 70% B in 2 min, then to 100% B in 0.2 min, which was held for 1 min. Re-equilibration took 5 min. ESI operating conditions were drying gas (N_2_, purity > 98%) at 350 °C and 10 L min^−1^; capillary voltage 4.0 KV; nebulizer gas 35 psig; sheath gas (N_2_, purity > 98%) at 375 °C and 12 L min^−1^. The fragmentor was kept at 175 V, and the collision energy voltage for the MS/MS experiments was set at 30 V. The collision gas used was nitrogen (purity 99.999%). Data were collected by auto MS/MS acquisition with an MS scan rate of 1.03 spectra sec^−1^, and only one precursor was acquired per cycle (relative threshold 0.010%). High-resolution MS and MS/MS spectra were achieved both in positive and negative mode in the range 100–1000 *m*/*z*; the mass axis was calibrated daily using the Agilent HP0321 tuning mix prepared in acetonitrile and water. Calibration curves were obtained for carthamin by analyzing the working solutions prepared in the 0.1–10 μg g^−1^ range in triplicate and by integrating the corresponding peaks at 520 nm. LOD was 0.04 μg g^−1^ and LOQ 0.1 μg g^−1^. Further details are reported in the Supplementary Materials (1. Supplementary Methods). Due to the lack of pure analytical standards, the relative amount of each quinochalcone in safflower samples was evaluated using a semi-quantitative approach on the basis of each chromatographic area integrated at 400 nm, normalized towards extract weight (EW, g) and dry sample weight (DW, g). Since different compounds have different molar absorptivity, our comparisons should be used to compare samples with one another, but not to compare the absolute quantities of different compounds within the same sample. Replicated analyses, performed on different aliquots of dried petals from a spring-sown Boemondo sample, showed an RSD lower than 10% for all the investigated analytes. Integration data, sample weight, and dilution factors are reported in the Supplementary Materials (3. Supplementary Tables, Tables S1 and S2).

*Normalized chromatographic peak area* = *chromatographic peak area* × *EW*/*DW*

For the analysis of red and yellow pigments, some milligrams of powdered safflower petals were placed in a pulverisette 23 ball mill (Fritsch, Germany) and subjected to an optimized extraction. In particular, 40 mg of each sample was inserted into a V-shaped extraction vial and added with 1 mL of MeOH, and the yellow components were extracted at 60 °C for 60 min in an ultrasonic bath. The supernatant was recovered in a separate vial while 1 mL of dimethylformamide was added to the residue; carthamin was extracted at 60 °C for 60 min in an ultrasonic bath. The MeOH solution containing the yellow compounds was dried, and then reconstituted with the DMF solution containing carthamin. The final solution containing both yellow compounds and carthamin was filtered on a 0.45 μm PTFE syringe filter and stored in the dark in a refrigerator at −20 °C until analyzed. All solutions were diluted c.a. 10 folds to obtain peak areas in the linear dynamic range of the instrument (3. Supplementary Tables, Tables S1–S3).

### 4.5. Extraction Methodology

A mass of 0.5 g of powdered safflower petals was placed in 250 mL Erlenmeyer flasks, to which 50 mL 70% (*v*/*v*) ethanol was added; they were then sonicated for 30 min at 60 °C. Then, samples were filtered through filter paper and stored at −20 °C until subsequent total phenol, total flavonoid, and antioxidant activity determinations.

### 4.6. Determination of Total Phenolic Contents

Determination of total phenolic compounds was performed on ethanolic extracts using the Folin–Ciocalteu method according to Dewanto et al. [64]. Quantification was performed using a calibration curve with gallic acid as standard, and the results were expressed as mg of gallic acid equivalents (GAE) per g of dry weight (DW).

### 4.7. Determination of Total Flavonoid Content

Total flavonoid content was determined according to the procedure described by Barros et al. [65] based on the formation of a flavonoid–aluminum complex. Quantification was performed using a calibration curve with rutin as standard, and the results were expressed as mg of rutin equivalents (RE) per g of dry weight (DW).

### 4.8. Ferric-Reducing Antioxidant Power (FRAP) Assay

Total antioxidant activity was measured by the FRAP method, according to Benzie and Strain (1996) [66] with slight modifications, as reported by Tavarini and Angelini [67]. This method measures the antioxidant capacity of a sample by evaluating its ability to reduce ferric ions (Fe^3+^) to ferrous ions (Fe^2+^) in a redox reaction, with the degree of color change indicating the level of antioxidant activity. In detail, the complex 2,4,6-tripyridyl-*S*-triazine (TPTZ)-Fe^3+^ is reduced to TPTZ-Fe^2+^ (colored form) in the presence of antioxidants. Quantification was performed using a calibration curve with ferrous sulfate as standard, and the results were expressed as μmol Fe^2+^ g^−1^ DW.

### 4.9. DPPH Radical-Scavenging Assay

Free radical-scavenging activity of safflower petal extracts was evaluated using the DPPH assay according to the method of Tadhani et al. [68]. This method is based on the reduction in the 2,2-diphenyl-1-picrylhydrazyl (DPPH) radical solution by antioxidants. This radical solution has a deep violet color, and, upon reduction, its color intensity decreases. As a result, an inverse relationship is observed between absorbance and antioxidant concentration. Radical-scavenging activity was calculated as inhibition of the free radical by the sample using the following formula:

% *inhibition* (% *I*) = [(*A*0 − *At*)/*A*0] × 100

where A0 is the absorbance of the control DPPH solution at 0 min and At is the absorbance of the extract after t = 20 min. The extract concentration providing 50% of the radical-scavenging activity (IC_50_) was calculated from the graph of inhibition percentage against extract concentration. The results were also expressed as ascorbic acid equivalents using a calibration curve with ascorbic acid as standard.

### 4.10. Statistical Analysis

All data were subjected to analysis of variance (ANOVA) using CoStat Version 6.2 (CoHort Software, Monterey, CA, USA). A two-way randomized blocks was carried out, with genotype (G), sowing time (S), and their interaction (G × S) as fixed, and block as random, to assess their influence on productivity (in terms of floret dry yield per head, head number per plant, and floret dry yield per plant) and quality (in terms of total phenols, flavonoids, and antioxidant activity). Means were separated on the basis of least significant difference (LSD) only when the ANOVA F-test showed significance at *p* ≤ 0.05. Block effect was not significant at *p* ≤ 0.05 for all traits considered. Principal Component Analysis (PCA) and Hierarchical Cluster Analysis (HCA) were performed on phytochemical analyses (red and yellow components, total phenols, total flavonoids, and antioxidant activity measured by FRAP and DPPH) using R Statistical Software (RStudio v1.4.1106, Boston, MA, USA). As unsupervised methods, the groups of samples obtained with both PCA and HCA analyses can be observed even when there are no reference samples that can be used as a training set to establish the model. The PCA was carried out on the correlation matrix with the goal of reducing the dimensionality of the multivariate matrix data (12 samples × 9 variables) whilst preserving most of the variance. Prior to Principal Component Analysis (PCA), means of all genotypes and sowing times for each variable have been used and subsequently centered and scaled. Since lower DPPH IC_50_ values indicate higher radical-scavenging activity, DPPH IC_50_ data were transformed using a reciprocal transformation (1/IC_50_) so that higher transformed values correspond to higher antioxidant activity. The number of principal components (PCs) was identified according to different criteria: the Kaiser–Guttman criterion (eigenvalues > 1), the percentage of variance explained cumulatively, and a scree and elbow plot. Variable weights/loadings were examined to identify the variables that contributed the most to each selected PC. The HCA was conducted on the normalized average values with Ward’s algorithm, using Euclidean distances as a measure of (dis)similarity among the samples. Before applying the hierarchical clustering method to assess whether the data were clusterizable, the Hopkins statistics (H) were used; a 0.63 H value was obtained, indicating a good propensity of our data to clusterize. If H < 0.5, the dataset is unlikely to have statistically significant clusters [69,70]. The result was a hierarchically clustered heatmap performed on the standardized data; this is also called a false-colored image, in which the data values were transformed into a color scale, showing the similarity among groups based on the red and yellow components, as well as the total phenols, total flavonoids, and antioxidant activity.

## 5. Conclusions

Our study highlighted that the genotype and sowing time significantly influenced the productive and qualitative traits of safflower crops. Among the tested genotypes, a heterogeneous behavior was observed in terms of both productive and biochemical parameters. The Pieve genotype showed the highest agronomic performances in terms of the floret dry yield per head, number of heads per plant, and floret dry yield per plant, while the Montola 2000 genotype was characterized by the highest polyphenol and flavonoid content, revealing the highest antioxidant activity and scavenging capacity. The sowing timing also emerged as a crucial factor in shaping the flowers’ phytochemical profiles: the autumn-sown florets showed a higher polyphenol content and antioxidant capacity compared to those sown in spring. Finally, these findings further emphasized the importance of considering genotype selection depending on the intended end-use of safflower, as genotypes differ significantly not only in flower yield but also in the quality profiles of key secondary metabolites. Therefore, it is necessary to implement targeted breeding programs for safflower aimed at optimizing agronomic and qualitative performance, with selection strategies explicitly adapted to the target value chain, whether it be the production of natural dyes or extracts enriched with bioactive compounds for nutraceutical applications. This approach will support the efficient valorization of safflower across different value chains and increase its potential as a multifunctional crop in the Mediterranean environment.

## Figures and Tables

**Table 1:** Effect of genotype, sowing date, and their reciprocal interaction on floret dry yield per head, head number per plant, and floret dry yield per plant.

Genotype	Floret Dry Yield Per Head (g)		Mean	No. Heads Per Plant		Mean	Floret Dry Yield Per Plant (g)		Mean
	Autumn	Spring		Autumn	Spring		Autumn	Spring	
Pieve	0.15 ± 0.05	0.16 ± 0.02	0.16 A	16.98 ± 3.75	15.75 ± 1.04	16.37 A	2.31 ± 0.19	2.51 ± 0.39	2.41 A
Boemondo	0.12 ± 0.02	0.13 ± 0.00	0.13 B	11.18 ± 1.31	8.83 ± 0.45	10.01 CD	1.30 ± 0.23	1.13 ± 0.18	1.22 CD
Belisario	0.11 ± 0.01	0.15 ± 0.02	0.13 B	11.78 ± 0.31	8.30 ± 1.15	10.04 CD	1.34 ± 0.03	1.22 ± 0.33	1.28 BC
Benno	0.11 ± 0.03	0.13 ± 0.02	0.12 B	11.06 ± 1.69	9.86 ± 1.94	10.46 C	1.21 ± 0.11	1.29 ± 0.33	1.25 BCD
Roberto	0.12 ± 0.02	0.14 ± 0.03	0.13 B	8.14 ± 0.96	8.33 ± 1.81	8.24 D	0.96 ± 0.11	1.12 ± 0.19	1.04 D
Montola 2000	0.09 ± 0.01	0.14 ± 0.01	0.12 B	14.36 ± 1.85	11.90 ± 2.96	13.13 B	1.29 ± 0.11	1.65 ± 0.54	1.47 B
Mean	0.12 B	0.14 A		12.25 A	10.50 B		1.40	1.49	

Mean values (±sd, n = 3) followed by different upper letters are statistically different for *p* ≤ 0.05 according to Fisher’s LSD test.

**Table 2:** Identified compounds along with their spectroscopic and spectrometric characteristics.

Peak No.	Identified Compound	Retention Time (min)	λ_max_ (nm)	Molecular Ion Pos. Mode	Main Product Ions (*m*/*z*)	Molecular Ion Neg. Mode	Main Product Ions (*m*/*z*)
1	hydroxysafflor yellow A (C_27_H_32_O_16_)	4.7	402, 237	[M + K]^+^ 651.13	633.11, 615.10, 531.08	[M − H]^−^ 611.16	491.12, 473.10
2	flavonoid glucoside (C_27_H_30_O_16_)	7.6	336	[M + H]^+^ 611.15	n.d.	[M − H]^−^ 609.14	449.10, 284.03, 255.03, 151.00
3	flavonoid glucoside (C_27_H_30_O_16_)	8.1	340	[M + H]^+^ 611.15	313.05, 303.04, 287.05, 211.02	[M − H]^−^ 609.14	300.02, 271.02, 151.00
4	safflor yellow A (C_27_H_30_O_15_)	15.4	407, 273	[M + H]^+^ 595.16	287.05	[M − H]^−^ 593.15	285.04
5	safflomin C and isomer (C_30_H_30_O_14_)	18.1	401	[M + H]^+^ 615.16	453.11, 289.06, 123.04	[M − H]^−^ 613.15	361.10, 287.05, 241.04, 119.05
6		18.7	406				
7	Carthamin (C_43_H_42_O_22_)	22.8	519, 380	[M + Na]^+^ 933.19	n.d.	[M − H]^−^ 909.20	502.10, 407.09, 287.05

All tandem mass spectra are reported in the Supplementary Materials (2. Supplementary Figures, Figures S7–S13).

**Table 3:** Chromatographic peak areas of quinochalcone compounds identified in each genotype sown in autumn and spring (C * = safflomin C isomer). Integration wavelength is reported in brackets. Areas were normalized towards extract volume and sample weight, and they are expressed in millions (normalized chromatographic peak area = chromatographic peak area x weight of the extracting solution (g)/sample dry weight (g)).

Genotype	Sowing Time	Normalized Chromatographic Peak Areas (400 nm) ± sd			
		Hydroxysafflor Yellow A	Safflor Yellow A	Safflomin C + C *	Total Quinochalcones
Pieve	autumn	1022 ± 76	792 ± 56	101 ± 8	1916 ± 140
	spring	1286 ± 96	1137 ± 81	96 ± 7	2519 ± 184
Boemondo	autumn	1030 ± 77	835 ± 59	100 ± 7	1965 ± 143
	spring	728 ± 54	526 ± 37	75 ± 6	1329 ± 97
Belisario	autumn	832 ± 62	525 ± 37	69 ± 5	1426 ± 104
	spring	1270 ± 95	939 ± 67	87 ± 7	2296 ± 167
Benno	autumn	2349 ± 176	1732 ± 123	253 ± 19	4334 ± 316
	spring	1086 ± 81	883 ± 63	94 ± 7	2064 ± 151
Roberto	autumn	1130 ± 84	956 ± 68	100 ± 7	2185 ± 159
	spring	896 ± 67	752 ± 53	77 ± 6	1725 ± 126
Montola 2000	autumn	998 ± 75	841 ± 60	107 ± 8	1946 ± 142
	spring	816 ± 61	691 ± 49	78 ± 6	1585 ± 116

**Table 4:** ANOVA table with F-values and statistical significance for total phenols, total flavonoids, and antioxidant activity (determined by FRAP assay) of safflower petal extracts, considering genotype (G), sowing time (S), and their mutual interaction (G × S) as fixed variability factors and block as random.

Source of Variation	Total Phenols	Total Flavonoids	FRAP	DPPH
Genotype (G)	17.82 ***	36.85 ***	22.30 ***	2277 ***
Sowing time (S)	32.75 ***	0.07 ns	23.36 ***	261.1 ***
G × S Block	16.94 *** 1.36 ns	4.54 ** 0.35 ns	12.51 *** 0.85 ns	1945 *** 44.92 ns

Results are based on Fisher’s LSD test: ns, not significant; ** significant at *p* ≤ 0.01; *** significant at *p* ≤ 0.001.

**Table 5:** Effect of genotype (G), sowing time (S), and their interaction (G × S) on total phenolic content (mg GAE g^−1^ DW) in safflower florets.

Genotype	Autumn Sowing	Spring Sowing	Mean
Pieve	25.56 ± 3.63 ab	13.58 ± 0.64 fg	19.57 BC
Boemondo	12.02 ± 2.78 g	17.11 ± 1.75 de	14.57 D
Belisario	24.62 ± 1.08 ab	15.68 ± 2.38 ef	20.15 BC
Benno	17.36 ± 0.85 dc	19.42 ± 0.42 cd	18.39 C
Roberto	23.66 ± 3.04 ab	19.68 ± 1.49 cd	21.67 B
Montola 2000	26.49 ± 0.50 a	22.34 ± 0.11 bc	24.42 A
Mean	21.62 A	17.97 B	

Means (±sd) with the same letters are not significantly different at *p* ≤ 0.05, according to Fisher’s LSD test. Genotype (G) and sowing time (S) are the variability factors. Upper-case letter: effect of genotype (G) and sowing date (S); lower-case letter: G × S interaction.

**Table 6:** Effect of genotype (G), sowing time (S), and their interaction (G × S) on total flavonoids (mg rutin equation g^−1^ DW) in safflower florets.

Genotype	Autumn Sowing	Spring Sowing	Mean
Pieve	4.88 ± 0.68 c	3.63 ± 0.49 e	4.26 BC
Boemondo	3.13 ± 0.23 e	3.50 ± 0.49 e	3.32 D
Belisario	3.55 ± 0.05 e	4.01 ± 0.34 cde	3.78 CD
Benno	3.85 ± 0.30 de	3.39 ± 0.84 e	3.62 D
Roberto	4.85 ± 0.62 c	4.59 ± 0.46 cd	4.72 B
Montola 2000	6.18 ± 0.81 b	7.59 ± 0.40 a	6.89 A
Mean	4.41 A	4.45 A	

Means (±sd) with the same letters are not significantly different at *p* ≤ 0.05, according to Fisher’s LSD test. Genotype (G) and sowing time (S) are the variability factors. Upper-case letter: effect of genotype (G) and sowing time (S); lower-case letter: G × S interaction.

**Table 7:** Effect of genotype (G), sowing time (S), and their interaction (G × S) on total antioxidant activity (µmol Fe^2+^ g^−1^ DW) in safflower florets measured using the FRAP assay.

Genotype	Autumn Sowing	Spring Sowing	Mean
Pieve	122.53 ± 16.20 ab	68.67 ± 3.13 e	95.60 BC
Boemondo	77.00 ± 16.43 e	94.40 ± 4.33 d	85.70 C
Belisario	123.22 ± 14.13 ab	70.58 ± 2.05 e	96.90 B
Benno	92.22 ± 4.09 d	105.97 ± 6.99 cd	99.10 B
Roberto	96.96 ± 0.03 d	105.18 ± 3.30 cd	101.07 B
Montola 2000	130.00 ± 3.72 a	115.28 ± 2.25 bc	122.64 A
Mean	106.98 A	93.34 B	

Means (±sd) with the same letters are not significantly different at *p* ≤ 0.05, according to Fisher’s LSD test. Genotype (G) and sowing time (S) are the variability factors. Upper-case letter: effect of genotype (G) and sowing time (S); lower-case letter: G × S interaction.

**Table 8:** DPPH radical-scavenging activity (half-inhibitory concentration, IC_50_, mg mL^−1^) of safflower floret extracts.

Genotype	Pieve	Boemondo	Belisario	Benno	Roberto	Montola 2000	Mean
Autumn sowing	2.25 ± 0.01 ^f^	3.20 ± 0.02 ^ab^	2.05 ± 0.01 ^g^	3.00 ± 0.01 ^b^	2.48 ± 0.02 ^d^	1.69 ± 0.01 ^h^	2.44 B
Spring sowing	3.21 ± 0.03 ^a^	2.47 ± 0.02 ^d^	2.60 ± 0.01 ^c^	2.43 ± 0.03 ^e^	2.49 ± 0.02 ^d^	2.06 ± 0.01 ^g^	2.54 A
Mean	2.73 B	2.83 A	2.32 D	2.71 B	2.48 C	1.87 E	
BHT	0.41						
Ascorbic Acid	0.16						
Trolox	0.02						

Means (±sd) with the same letters are not significantly different at *p* ≤ 0.05, according to Fisher’s LSD test. Genotype (G) and sowing time (S) are the variability factors. Upper-case letter: effect of genotype (G) and sowing time (S); lower-case letter: G × S interaction.

## Data Availability

The original contributions presented in this study are included in the article/Supplementary Material. Further inquiries can be directed to the corresponding author.

## Supplementary Materials

The supporting information can be downloaded at: https://www.mdpi.com/article/10.3390/plants15020282/s1.

## Abbreviations

The following abbreviations are used in this manuscript:

ACN	Acetonitrile
BHT	Butylated Hydroxytoluene
DAD	Diode Array Detector
DMF	Dimethylformamide
DPPH	2,2-Diphenyl-1-picrylhydrazyl
ESI	Electrospray Ionization
EtOH	Ethanol
FRAP	Ferric Reducing Antioxidant Power
GAE	Gallic Acid Equivalent
H_2_O	Water
HPLC	High-Performance Liquid Chromatography
HPLC-ESI-Q-ToF	High-Performance Liquid Chromatography–Electrospray Ionization–Quadrupole Time-of-Flight
IC_50_	Half-Maximal Inhibitory Concentration
LC-MS	Liquid Chromatography–Mass Spectrometry
LOD	Limit of Detection
LOQ	Limit of Quantification
MeOH	Methanol
MS/MS	Tandem Mass Spectrometry
RE	Rutin Equivalent
TFA	Trifluoroacetic Acid
UV–Vis	Ultraviolet Visible
