# Supplementary material for: Natural Dyes and Antioxidant Compounds from Safflower (Carthamus tinctorius L.) Florets: The Effects of Genotype and Sowing Time
Source: Plants (Basel). 2026 Jan 17;15(2):282. doi: 10.3390/plants15020282 (PMC12844731; doi:10.3390/plants15020282)
Supplement: Supplementary file 1 [file plants-15-00282-s001.zip › plants-3999463-supplementary.pdf]

Supplementary Material

Natural Dyes and Antioxidant Compounds from Safflower (*Carthamus tinctorius* L.) Florets: The Effects of Genotype and Sowing Time

Clarissa Clemente [1], Silvia Tavarini [1,2,*], Shaula Antoni [3], Silvia Zublena [4], Luciana G. Angelini [1,2] and Ilaria Degano [3]

[1] Department of Agriculture, Food and Environment, University of Pisa, Via del Borghetto 80, 56124 Pisa, Italy; clarissa.clemente@agr.unipi.it (C.C.); luciana.angelini@unipi.it (L.G.A.)
[2] Interdipartimental Research Center "Nutraceuticals and Food for Health", University of Pisa, Via del Borghetto 80, 56124 Pisa, Italy
[3] Department of Chemistry and Industrial Chemistry, University of Pisa, Via Moruzzi, 13, 56124 Pisa, Italy; shaulaantoni@gmail.com (S.A.); ilaria.degano@unipi.it (I.D.)
[4] Pisa Botanic Garden and Museum, University of Pisa, Via Luca Ghini 13, I, 56126 Pisa, Italy; silvia.zublena@unipi.it
* Correspondence: silvia.tavarini@unipi.it; Tel.: +39-0502218948

Table of contents

1. Supplementary Methods 2

1.1. Quantitation of carthamin via HPLC-DAD method 2

2. Supplementary Figures 2

2.1. Representative HPLC-DAD chromatograms of the extracts from the different genotypes and different sowing times 2

2.2. Tandem mass spectra of the compounds identified in the extracts 5

3. Supplementary Tables 9

3.1. Data used for quantitation and semi-quantitation by HPLC-DAD analysis. 9

3.2. PCA variables and loadings 10

1. Supplementary Methods

1.1. Quantitation of carthamin via HPLC-DAD method

Calibration curves were obtained for carthamin by analyzing the working solutions prepared in the 0.1-10 μg g^{-1} range (concentration levels were 0. 1; 0.3; 0.7; 1.2; 5.0; 12.6 μg g^{-1}) in triplicate and by integrating the corresponding peaks at 520 nm. The equation of the calibration curve was Area = 2.1×10^4 [carthamin/ μg g^{-1}] + 6.3×10^2, $R^2 = 0.9999$; LOD was 0.04 μg g^{-1} and LOQ 0.1 μg g^{-1}, calculated as the concentration corresponding to a signal equal to the 3 and 10 times the standard deviation of the blanks.

2. Supplementary Figures

2.1. Representative HPLC-DAD chromatograms of the extracts from the different genotypes and sowing times

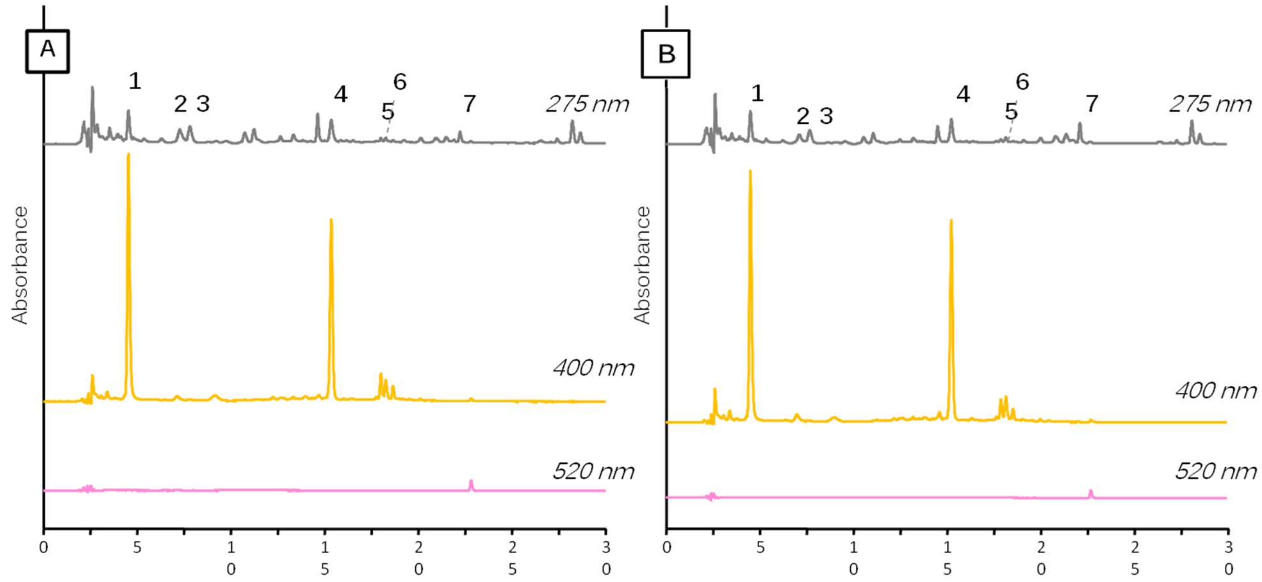

Figure S1. Chromatograms at 275, 400, and 520 nm of the extract derived from the Pieve samples sown in autumn (A, on the left) and in spring (B, on the right). Peak 1: hydroxysafflor yellow A; peaks 2 and 3: flavonoid glucosides; peak 4: safflor yellow A; peak 5: safflomin C; peak 6: isomer of safflomin C; peak 7: carthamin. Chromatograms are presented in the same scale and staked for clarity.

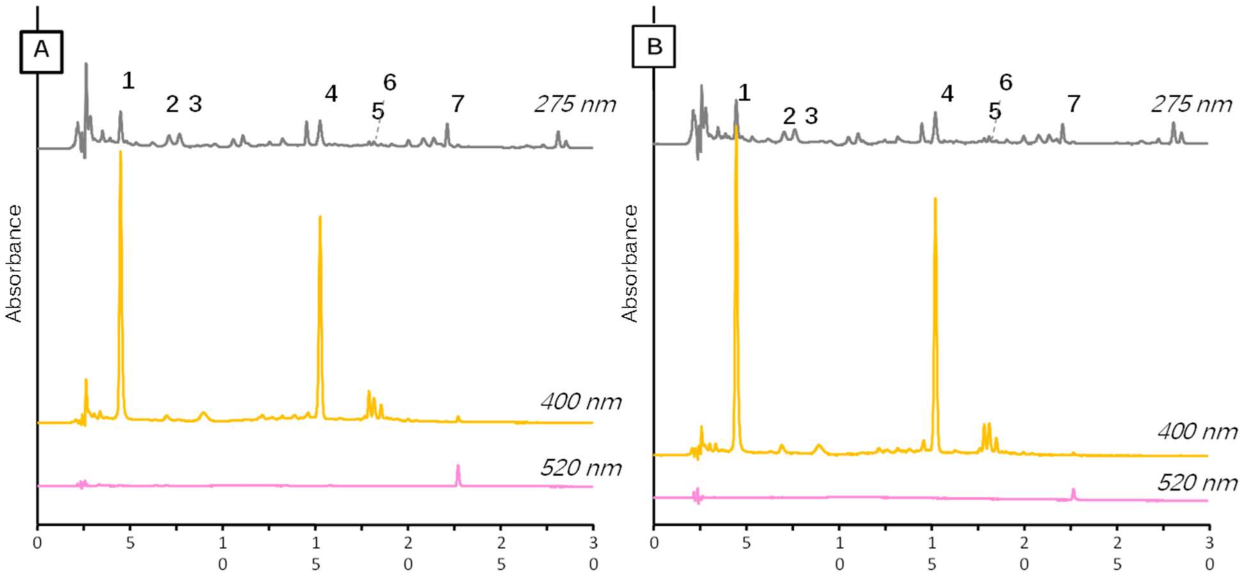

Figure S2. Chromatograms at 275, 400, and 520 nm of the extract derived from the Boemondo samples sown in autumn (A, on the left) and in spring (B, on the right). Peak 1: hydroxysafflor yellow A; peaks 2 and 3: flavonoid glucosides; peak 4: safflor yellow A; peak 5: safflomin C; peak 6: isomer of safflomin C; peak 7: carthamin. Chromatograms are presented in the same scale and staked for clarity. [The chromatograms on the left correspond to those shown in Figure 2 in the main text]

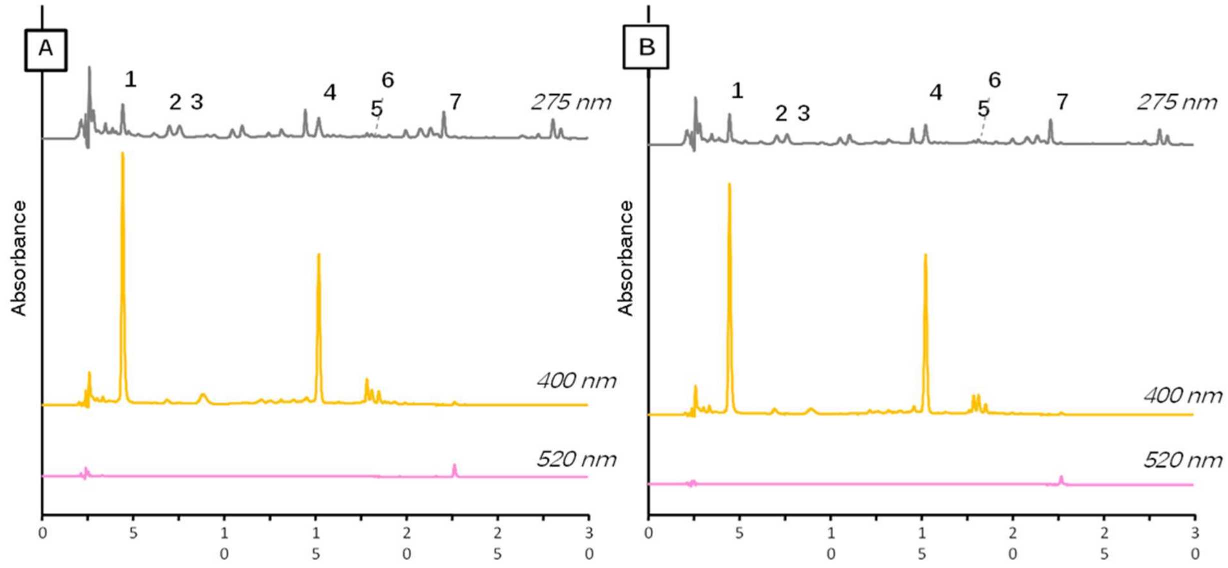

Figure S3. Chromatograms at 275, 400, and 520 nm of the extract derived from the Belisario samples sown in autumn (A, on the left) and in spring (B, on the right). Peak 1: hydroxysafflor yellow A; peaks 2 and 3: flavonoid glucosides; peak 4: safflor yellow A; peak 5: safflomin C; peak 6: isomer of safflomin C; peak 7: carthamin. Chromatograms are presented in the same scale and staked for clarity.

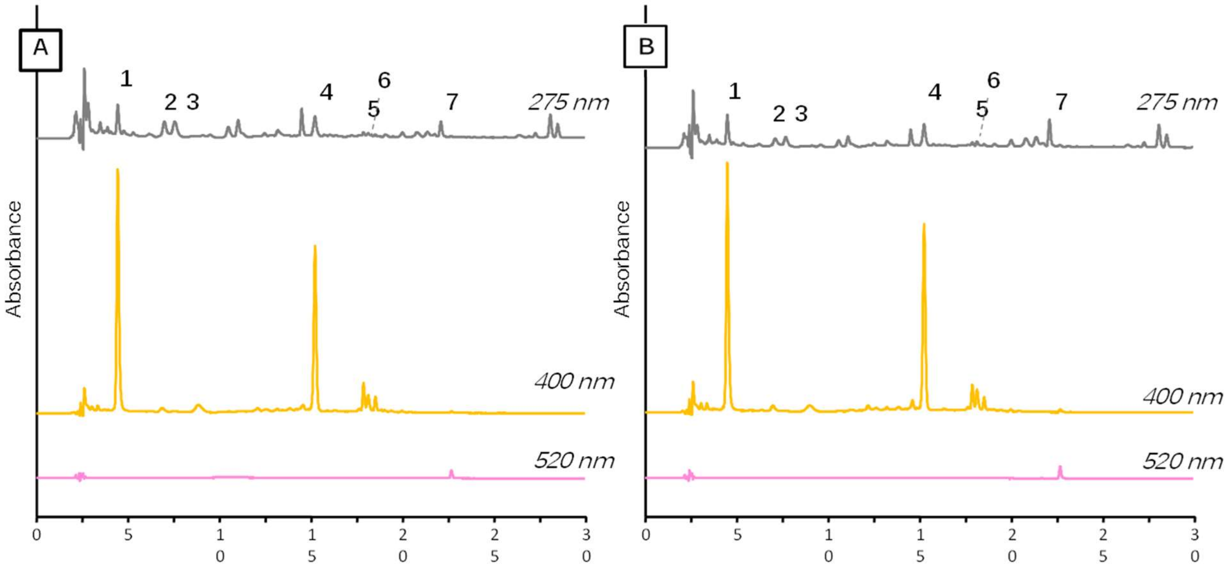

Figure S4. Chromatograms at 275, 400, and 520 nm of the extract derived from the Benno samples sown in autumn (A, on the left) and in spring (B, on the right). Peak 1: hydroxysafflor yellow A; peaks 2 and 3: flavonoid glucosides; peak 4: safflor yellow A; peak 5: safflomin C; peak 6: isomer of safflomin C; peak 7: carthamin. Chromatograms are presented in the same scale and staked for clarity.

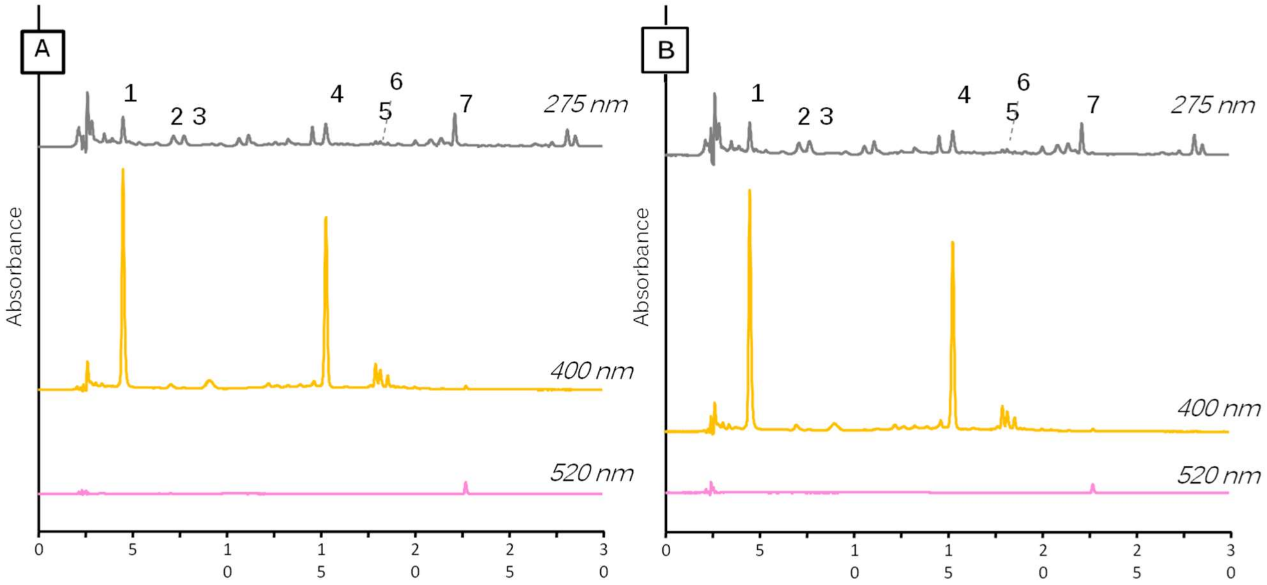

Figure S5. Chromatograms at 275, 400, and 520 nm of the extract derived from the Roberto samples sown in autumn (A, on the left) and in spring (B, on the right). Peak 1: hydroxysafflor yellow A; peaks 2 and 3: flavonoid glucosides; peak 4: safflor yellow A; peak 5: safflomin C; peak 6: isomer of safflomin C; peak 7: carthamin. Chromatograms are presented in the same scale and staked for clarity.

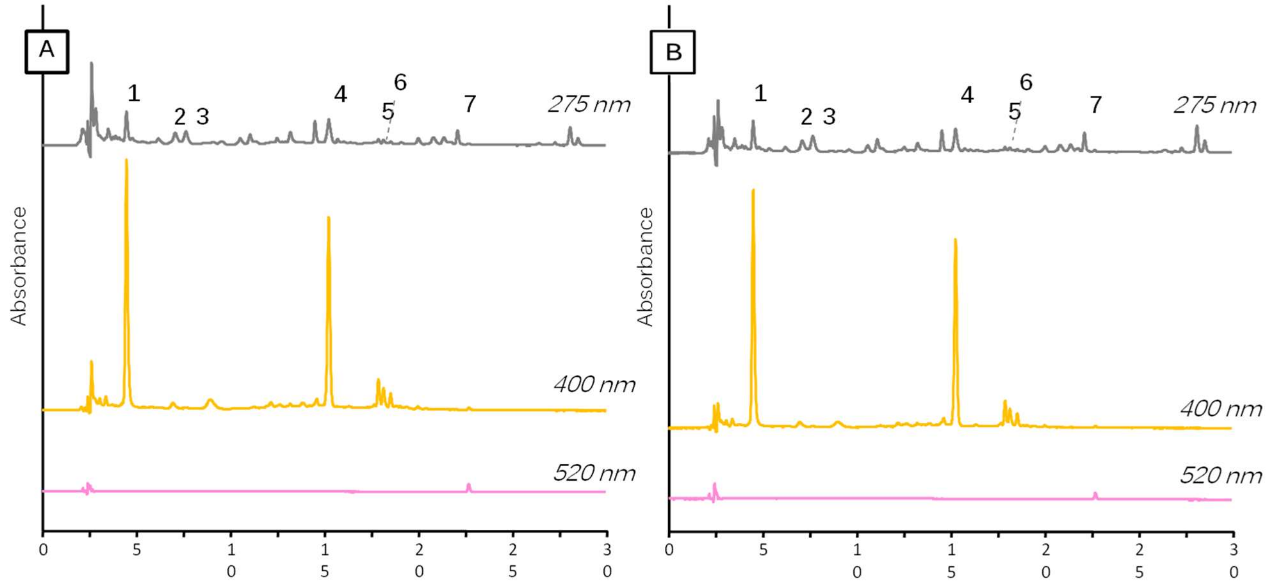

Figure S6. Chromatograms at 275, 400, and 520 nm of the extract derived from the Montola 2000 samples sown in autumn (A, on the left) and in spring (B, on the right). Peak 1: hydroxysafflor yellow A; peaks 2 and 3: flavonoid glucosides; peak 4: safflor yellow A; peak 5: safflomin C; peak 6: isomer of safflomin C; peak 7: carthamin. Chromatograms are presented in the same scale and staked for clarity.

2.2. *Tandem mass spectra of the compounds identified in the extracts*

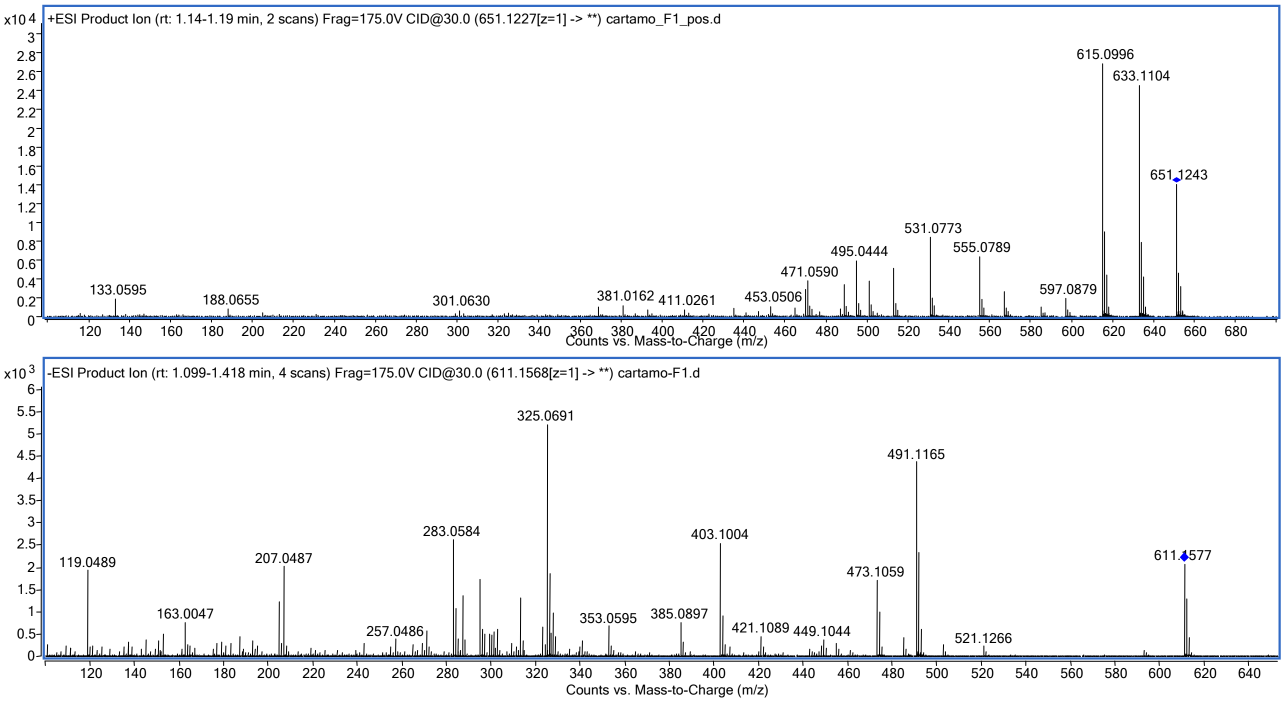

Figure S7. Tandem mass spectrum acquired in positive (top) and negative (bottom) ionization mode, collision cell voltage 30V, for the $[M+K]^+$ precursor ion at 651.1227 *m/z* and the $[M-H]^-$ precursor ion at 611.1568 *m/z* ($C_{27}H_{32}O_{16}$), respectively, attributed to hydroxysafflor yellow A (peak #1 in Table 2 and Figure 2 in the main text and in Figures S 1-6).

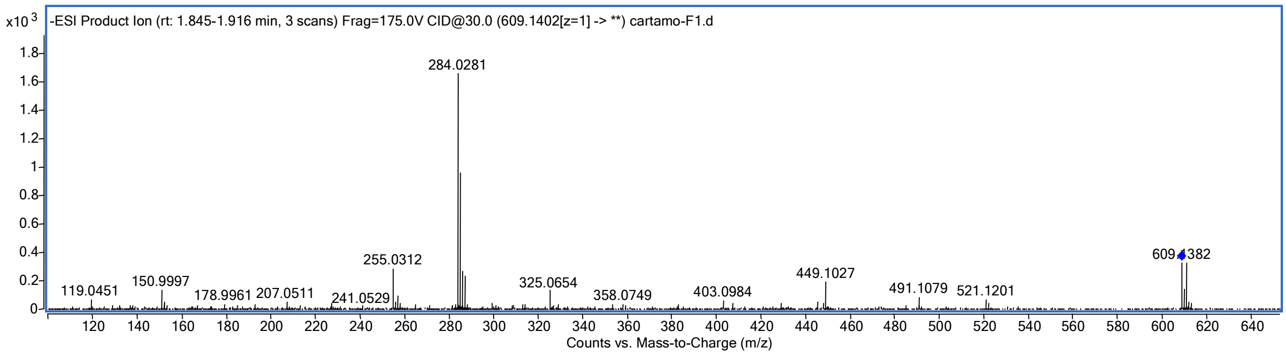

Figure S8. Tandem mass spectrum acquired in negative ionization mode, collision cell voltage 30V, for the precursor ion [M-H]$^-$ at 609.1402 *m/z* ($C_{27}H_{30}O_{16}$) attributed to a flavonoid glucoside (peak #2 in Table 2 and Figure 2 in the main text and in Figures S 1-6).

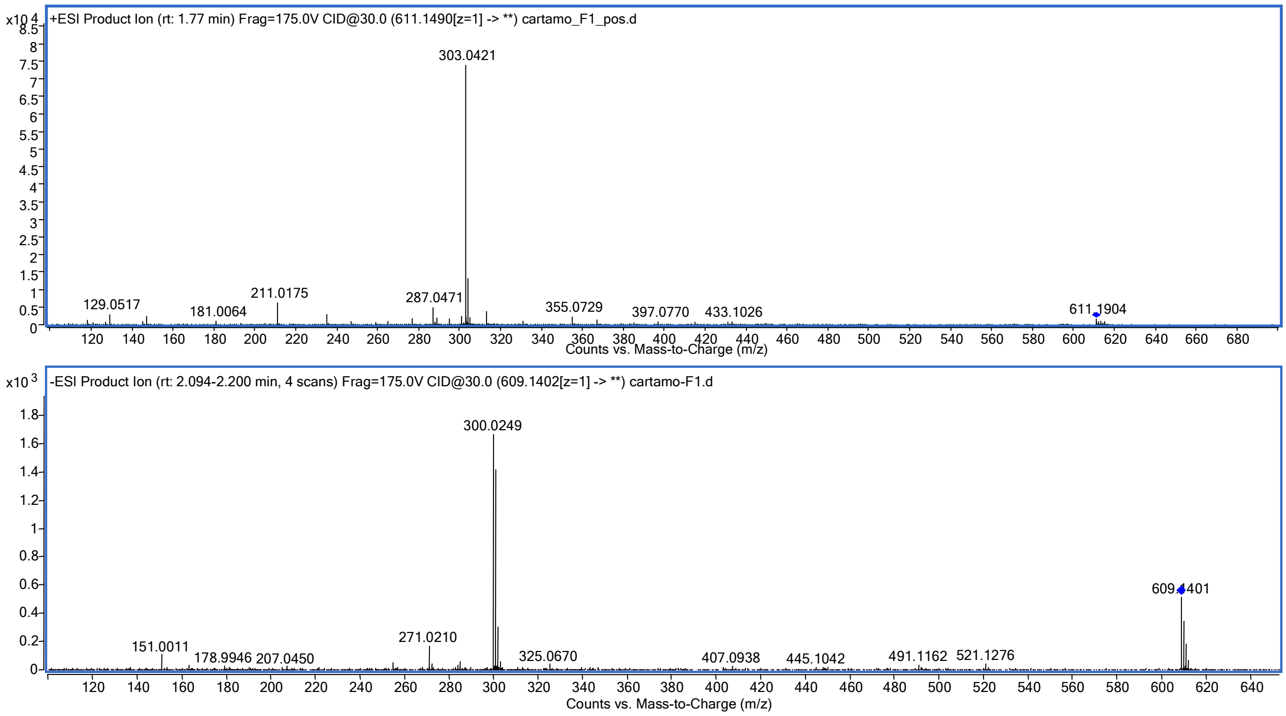

Figure S9. Tandem mass spectrum acquired in positive (top) and negative (bottom) ionization mode, collision cell voltage 30V, for the precursor ion [M+H]$^+$ at 611.1904 *m/z* and [M-H]$^-$ at 609.1402 *m/z* ($C_{27}H_{30}O_{16}$), respectively, attributed to a flavonoid glucoside (peak #3 in Table 2 and Figure 2 in the main text and in Figures S 1-6).

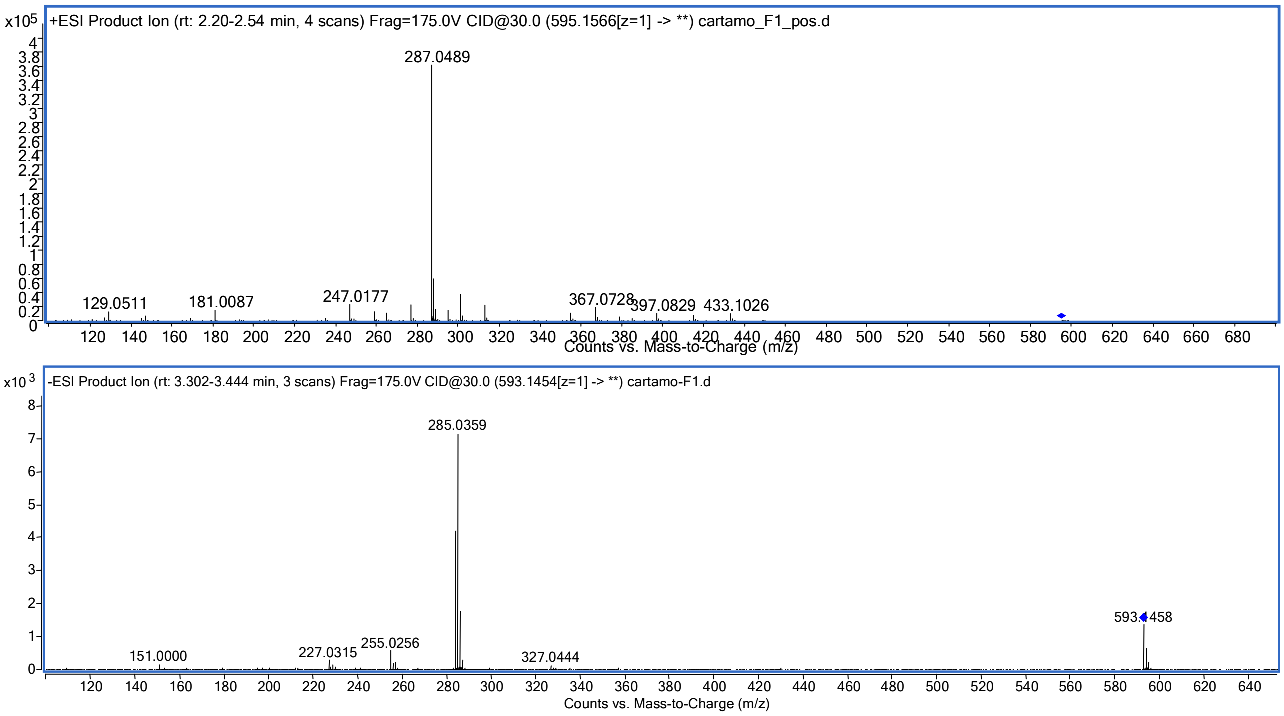

Figure S10. Tandem mass spectrum acquired in positive (top) and negative (bottom) ionization mode, collision cell voltage 30V, for the precursor ion $[M+H]^+$ at 595.1566 *m/z* and $[M-H]^-$ at 593.1454 *m/z* ($C_{27}H_{30}O_{15}$), respectively, attributed to safflor yellow A (peak #4 in Table 2 and Figure 2 in the main text and in Figures S 1-6).

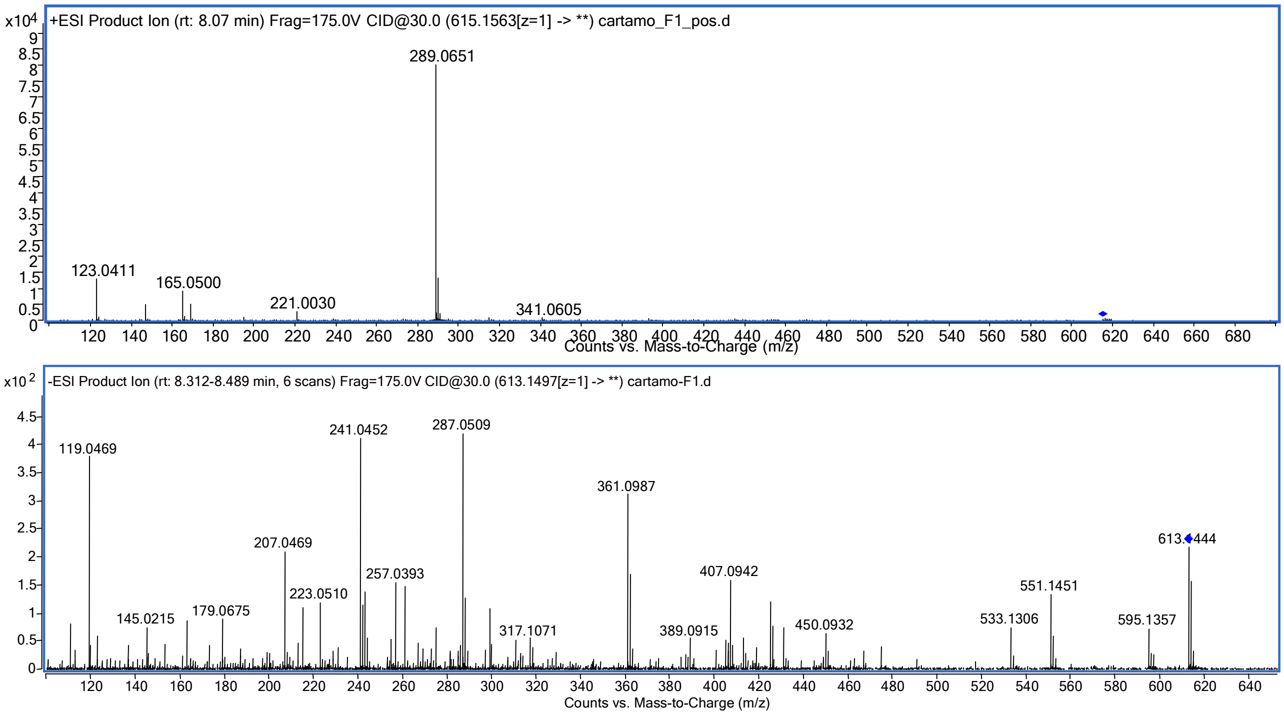

Figure S11. Tandem mass spectrum acquired in positive (top) and negative (bottom) ionization mode, collision cell voltage 30V, for the precursor ion $[M+H]^+$ at 615.1563 *m/z* and $[M-H]^-$ at 613.1497 *m/z* ($C_{30}H_{30}O_{14}$), respectively, attributed to safflomin C (peak #5 in Table 2 and Figure 2 in the main text and in Figures S 1-6).

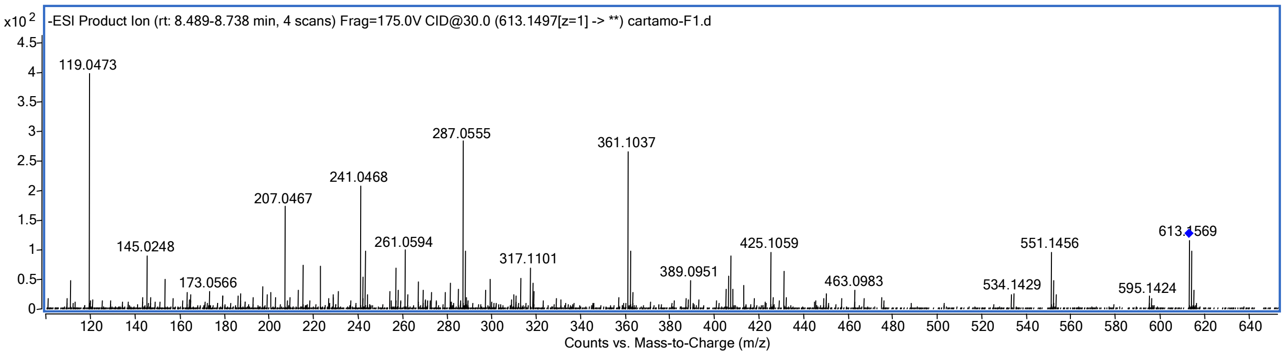

Figure S12. Tandem mass spectrum acquired in negative ionization mode, collision cell voltage 30V, for the precursor ion $[M-H]^-$ at 613.1497 *m/z* ($C_{30}H_{30}O_{14}$) attributed to safflomin C isomer (peak #6 in Table 2 and Figure 2 in the main text and in Figures S 1-6).

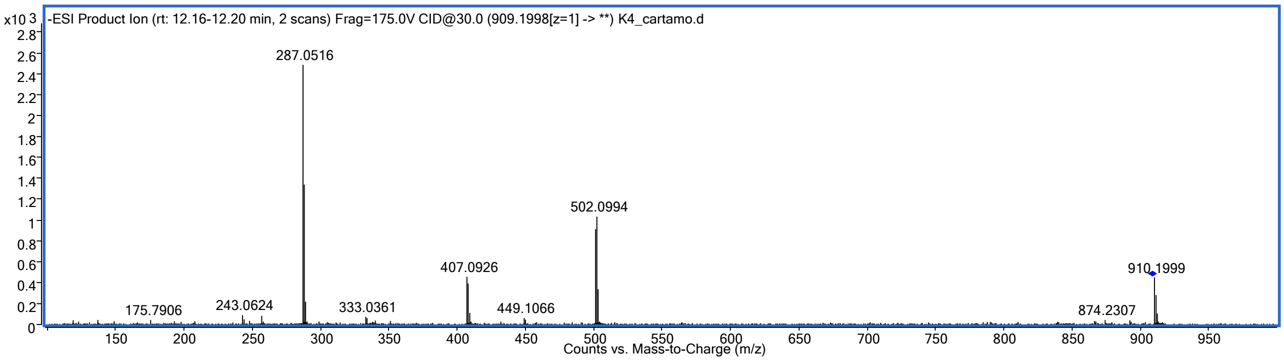

Figure S13. Tandem mass spectrum acquired in negative ionization mode, collision cell voltage 30V, for the precursor ion $[M-H]^-$ at 909.1998 *m/z* ($C_{43}H_{42}O_{22}$) attributed to carthamin (peak #7 in Table 2 and Figure 2 in the main text and in Figures S 1-6).

3. Supplementary Tables

3.1. Data used for quantitation and semi-quantitation by HPLC-DAD analysis

Table S1. Sample weight and dilution factors for all samples analysed by HPLC-DAD.

	autumn						spring							
	Pieve	Boemondo	Belisario	Benno	Roberto	Montola 2000	Pieve	Boemondo r1	Boemondo r2	Boemondo r3	Belisario	Benno	Roberto	Montola 2000
sample weight (g)	0.0415	0.0388	0.0500	0.0324	0.0416	0.0383	0.0390	0.0412	0.0359	0.0419	0.0439	0.0386	0.0458	0.0427
extract weight (g)	1.1591	1.0995	1.1964	1.0237	1.1311	1.2201	1.2220	1.3407	0.9423	1.3888	1.1695	1.2822	1.3988	1.4837
aliquot for dilution (g)	0.1059	0.0891	0.0865	0.0504	0.1072	0.0864	0.0885	0.1094	0.0896	0.0892	0.0889	0.0872	0.0858	0.0847
dilution solution (g)	1.0961	1.0423	1.0789	1.2484	1.0991	1.0813	1.0762	1.1015	1.0864	1.0823	1.0829	1.0840	1.0803	1.0792
dilution factor	10.3503	11.6981	12.4728	24.7698	10.2528	12.5150	12.1605	10.0686	12.1250	12.1334	12.1811	12.4312	12.5909	12.7414

Table S2. Integrated peak areas for HPLC-DAD analysis for all samples and compounds considered.

		Area													
		autumn						spring							
compound /genotype	integration wavelenght (nm)	Pieve	Boemon do	Belisario	Benno	Roberto	Montola 2000	Pieve	Boemon do r1	Boemon do r2	Boemon do r3	Belisario	Benno	Roberto	Montola 2000
hydroxysaff lor yellow A	400	3739972	3278540	3090454	2461666	4169789	2825104	3832380	2556411	2152516	2250983	4226503	3111362	3016420	2532502
Flv-glu1	275	43602	64510	47135	28193	106785	74201	128897	87946	75189	81347	104714	64730	73306	62900
Flv-glu2	275	91143	219731	246035	113711	382510	240724	91365	176209	157316	155374	106878	153550	143120	80079
safflor yellow A	400	2898442	2656699	1949346	1814483	3528735	2379832	3389414	1852314	1549061	1628802	3123383	2529324	2532660	2147211
safflomin C	400	245624	211722	174582	169154	222668	206418	200121	175928	142903	144976	183710	166303	169771	168740
safflomin C*	400	123554	105715	83630	96181	145737	96809	85806	98284	80846	82381	106228	104199	90187	73514
carthamin	520	132383	216314	121598	66887	177250	80132	98826	182563	135066	135017	118246	125817	93171	57343

3.2. PCA variables and loadings

Table S3. Loadings of variables for the first two principal components (PC1 and PC2) obtained from PCA.

Variable	PC1	PC2
Hydroxysafflor yellow A	**0.403**	0.229
Safflor yellow A	**0.402**	0.211
Safflomin C	**0.368**	0.321
Isosafflomin C	**0.377**	0.304
Carthamin	**0.364**	0.040
Total phenols	−0.244	**0.465**
Total flavonoids	−0.194	**0.385**
FRAP	−0.260	**0.440**
DPPH ($1/IC_{50}$)	−0.319	**0.383**

Loadings with higher absolute values indicate variables contributing most strongly to each principal component.
